# Clinical evaluation of advanced MALDI-TOF MS for carbapenemase subtyping in Gram-negative isolates

**DOI:** 10.1128/jcm.01475-24

**Published:** 2024-11-29

**Authors:** Dong Huey Cheon, Heejung Jang, Yoon Kyung Choi, Won Seok Oh, Seohyun Hwang, Ju-Ri Park, Hyojin Kim, Yoonha Park, Saeyoung Lee, Won Suk Yang, Min Jin Kim, Sun Hwa Lee, Je-Hyun Baek

**Affiliations:** 1 R&D Center for Clinical Mass Spectrometry, Seegene Medical Foundation 595499 , Seoul, Republic of Korea; 2 Department of Laboratory Medicine, Seegene Medical Foundation 595499 , Seoul, Republic of Korea; NorthShore University HealthSystem Department of Pathology and Laboratory Medicine 549656 , Evanston, Illinois, USA

**Keywords:** carbapenemase-producing Enterobacteriaceae, MALDI-TOF MS, carbapenemase, clinical diagnostics, antibiotics resistance, subtyping

## Abstract

The spread of carbapenemase-producing Enterobacterales (CPE) is emerging as a significant clinical concern in tertiary hospitals and, in particular, long-term care facilities with deficiencies in infection control. This study aims to evaluate an advanced matrix-assisted laser desorption/ionization (A-MALDI) mass spectrometry method for the identification of carbapenemases and further discrimination of their subtypes in clinical isolates. The A-MALDI method was employed to detect CPE target proteins. Enhancements were made to improve detectability and mass accuracy through the optimization of MALDI-TOF settings and internal mass calibration. A total of 581 clinical isolates were analyzed, including 469 CPE isolates (388 *Klebsiella pneumoniae* carbapenemases [KPC], 51 NDM, 40 OXA, and 2 GES) and 112 carbapenemase-negative isolates. Clinical evaluation of the A-MALDI demonstrated 100% accuracy and precision in identifying all the collected CPE isolates. Additionally, A-MALDI successfully discriminated individual carbapenemase subtypes (KPC-2 or KPC-3/KPC-4, OXA-48 or OXA-181 or OXA-232, GES-5 or GES-24) and also differentiated co-producing carbapenemase strains (KPC and NDM, KPC and OXA, KPC and GES, and NDM and OXA), attributed to its high mass accuracy and simultaneous detection capability. A-MALDI is considered a valuable diagnostic tool for accurately identifying CPE and carbapenemase’s subtypes in clinical isolates. It may also aid in selecting appropriate antibiotics for each carbapenemase subtype. Ultimately, we expect that the A-MALDI method will contribute to preventing the spread of antibiotic resistance and improving human public health.

**IMPORTANCE:**

A-MALDI clearly demonstrated excellent ability to identify CPEs such as KPC, NDM, OXA, and GES when carbapenemase is present in the strain (100% accuracy and precision). The method also successfully discriminated carbapenemase subtypes and simultaneous detection of co-producing multiple carbapenemases in a single strain. This is the first report for simultaneous and multiple detection of intact carbapenemases of KPC, NDM, OXA, and GES using matrix-assisted laser desorption/ionization mass spectrometry in a clinical isolate.

## INTRODUCTION

The emergence and dissemination of diseases caused by bacteria or viruses worldwide significantly threaten public health (1). In addition, the overuse of antibiotics has led to antibiotic-resistant strains of bacteria that are difficult to treat and cause severe illness (2). Antibiotic resistance has been acknowledged as a “silent pandemic” and has emerged as a critical public health concern that threatens human survival (3). Carbapenems have been widely used to treat bacterial infections; however, the emergence of carbapenem-resistant *Enterobacterales* (CRE) has severely limited their effectiveness (4).

The carbapenem resistance is primarily driven by four mechanisms: β-lactamase production, porin channel alterations, efflux pump activity, and penicillin-binding protein modifications (5). Among these resistance mechanisms, β-lactamase production is most critical. These enzymes can rapidly and efficiently degrade β-lactam antibiotics, including the most potent and broad-spectrum agents (6). Infections caused by carbapenemase-producing Enterobacterales (CPE) are associated with significantly higher mortality rates compared to those caused by non-CPE organisms (7). Furthermore, carbapenemase-encoding genes typically located on mobile genetic elements facilitate the global spread of CPE, making their carbapenem resistance highly transmissible (8).

Residents of long-term care facilities (LTCFs), such as nursing homes and long-term acute care hospitals, are particularly vulnerable to CRE infection and colonization (9). Several surveillance studies have shown that the prevalence of *Klebsiella pneumoniae* CPE is approximately nine times higher in long-term acute care hospitals than in acute care hospitals (10, 11). A study conducted in Korea demonstrated that between 2007 and 2018, the rates of antimicrobial resistance among isolates from LTCFs were also higher than those from other medical institutions (12).

Given the severe implications of CPE infections and transmission, differentiating carbapenemase variants in Enterobacterales is crucial for effective control and treatment strategies (13). This is particularly evident in the *Klebsiella pneumoniae* carbapenemase (KPC) family, which has more than 150 reported variants globally. New variants are emerging rapidly, particularly in regions such as the United States, China, and Europe (13). Accurate identification of these carbapenemase variants is essential for tracking resistant strains and guiding infection control measures. Such identification may help to reveal transmission patterns, allowing for more targeted efforts to contain outbreaks. Moreover, KPC variants exhibit heterogeneity, likely arising from diverse antibiotic exposure patterns (14). This effect is significant because it influences treatment strategies and the choice of antibiotics in clinical practice (14). An important aspect of understanding these variants is the “seesaw effect,” where mutations in KPC variants that confer resistance to one class of antibiotics, such as ceftazidime-avibactam, could simultaneously restore susceptibility to other antibiotics, particularly carbapenems like imipenem (13).

Various phenotypic, genotypic, and immunological methods have been developed for CPE detection. Culture-based methods (e.g., carbapenem inactivation methods) are cost effective and widely applicable in clinical settings. Colorimetric tests (e.g., CarbaNP) have also been developed as rapid, simple, and cost-effective methods. However, these phenotypic methods require additional culture steps or depend on bacterial growth rates, which did not provide any molecular evidence. Polymerase chain reaction (PCR) is one of the most efficient and widely utilized rapid molecular tools for quantifying and profiling genes, although it requires high cost and skilled technicians to perform the testing. Lateral-flow immunochromatographic tests (e.g., RESIST-4 O.K.N.V.) are rapid diagnostic tools based on antibody-antigen interactions, but the method often shows false-negative or lower detection sensitivity upon specific culture conditions (15). However, no clinical assay can discriminate their subtypes for CPE proteins or genes yet (16).

Matrix-assisted laser desorption/ionization mass spectrometry (MALDI-TOF MS) has emerged as a powerful tool in clinical microbiology, enabling the ionization of multiple proteins and measurement of their molecular mass according to the mass-to-charge ratio (*m*/*z*). This method enables the simultaneous detection of ribosomal proteins for identifying bacterial species (17). Furthermore, MALDI-TOF MS has shown potential in identifying simultaneously multiple carbapenemases by detecting intact carbapenemase peaks (18). To maximize its clinical utility, there is a need for an expanded MALDI-TOF method capable of detecting other carbapenemases beyond KPC-2, such as NDM, OXA, and GES.

We previously reported on MALDI-TOF MS analysis using osmotic shock (OS) lysis (OS-MALDI), a rapid, low-cost, and detergent-free method for detecting periplasmic proteins from Gram-negative bacteria (19). The OS-MALDI method showed outstanding accuracy, precision, specificity, and sensitivity in detecting intact KPC proteins in clinical isolates (18). Additionally, we demonstrated that internal mass calibration (In-Cal) process enhances mass accuracy for high-mass range targets in MALDI-TOF analysis (20). In this study, we introduce an advanced MALDI-TOF MS method, termed advanced matrix-assisted laser desorption/ionization (A-MALDI), developed by integration of our previous techniques. Moreover, we evaluate its efficacy in detecting carbapenemase-producing clinical isolates that originated from LTCFs and other hospitals.

## MATERIALS AND METHODS

### Sample collection and CPE classification

Clinical isolates were collected by Seegene Medical Foundation (Seoul, Republic of Korea) from 18 LTCFs (89%) and 15 other hospitals (11%). The isolates were cultured on a selective chromogenic medium (CHROM mSuperCARBA Agar; Asan Pharmaceutical, Republic of Korea) to detect CRE. Carbapenemase-positive isolates producing *bla*_KPC_, *bla*_NDM_, *bla*_OXA_, and *bla*_GES_ genes, confirmed by PCR and DNA sequencing, were classified as CPE. Isolates that did not grow on the medium, confirmed by negatives for these genes through PCR and DNA sequencing, were classified as carbapenemase negative. A total of 581 clinical isolates were evaluated in this study.

### Sample preparation for A-MALDI

Fifteen colonies per isolate, cultured on blood agar plate at 37°C for 16–20 h, were used for the A-MALDI. The selected colonies were suspended in 100 µL of 500-mM NaCl in 25-mM Tris-HCl (pH 8.0). The suspended cells were then incubated at RT for 10 min and centrifuged at 14,000 × *g* for 10 min. The supernatant was discarded, and 100 µL distilled water (DW) was added. Next, the cell pellet was resuspended in DW and incubated at RT for 10 min. After centrifugation, the supernatant was collected (i.e., OS sup) for the analysis of KPC, OXA, and GES proteins. The remaining pellet was subsequently resuspended in 50 µL of 20% (vol/vol) 2,2,2-trifluoroethanol (TFE, Sigma-Aldrich) and incubated at room temperature (RT) for 10 min. Five micrograms of SP HP resin (GE Healthcare), resuspended in 50 µL of DW, was added to the pellet. The colloidal sample was incubated at RT for 10 min with gentle vortexing. After centrifugation, the supernatant was collected (i.e., TFE sup). Finally, TFE sup was diluted to a final concentration of 1% (vol/vol) *n*-octyl-β-D-glucoside for the analysis of NDM proteins. For more information in details, refer to the Supplemental Material.

### MALDI-TOF MS analysis for A-MALDI

MALDI-TOF MS analysis was conducted using a Microflex LT/SH smart system (Bruker Daltonics GmbH & Co. KG, Germany), with laser frequency set to 100 Hz, a mass range of 12,000–32,000 *m*/*z*, ion source 1 and 2 voltages of 20 and 18 kV, respectively, and a lens voltage of 7 kV. External mass calibration was performed daily prior to sample analysis using purified KPC-2 protein. The MALDI-TOF MS parameters were flexibly adjusted daily before the intact protein analysis, including modulation of the absolute laser power (optimal values were 85%–92%) and the detector gain (optimal values were 2,381–3,083 V). Spectra were acquired using a random walk mode, with each spectrum comprising 1,000 laser shots, with different positions for each shot. MALDI spotting for A-MALDI was as follows: for OS sup analysis, 1 µL of a sample, 1 µL of internal calibrator (6xHis-tagged KPC-2 [hKPC-2]), and 1 µL of sinapinic acid (SA) were spotted and then mixed onto a MALDI-TOF MS target plate. After drying, MALDI-TOF MS analysis was performed. For TFE sup analysis, 1 µL of SA was spotted onto a clean MALDI-TOF MS target plate and allowed to dry. Subsequently, 1 µL of sample and 1 µL of internal calibrator were spotted onto the dried SA spot and allowed to dry. Another 1 µL of SA was then spotted onto the dried analyte spot. After drying, MALDI-TOF MS analysis was performed.

### In-Cal process of MALDI-TOF MS spectra

The recombinant hKPC-2 protein was used for In-Cal, as reported previously (20). For MALDI-TOF analysis, 60 ng of internal calibrator was spiked into each sample, and the data were processed using our in-house R package. In-Cal was performed using the singly and doubly charged ion peaks of the hKPC-2 protein in the mass spectra. Specifically, a linear function was generated by computing the difference between the theoretical value of the two hKPC-2 peaks and the corresponding *m*/*z* of the peak detected in the sample (Fig. S1). Subsequently, all peaks were internally calibrated using the function derived from the spectrum data of each sample.

## RESULTS

### A-MALDI: a brief overview

The A-MALDI represents an advanced MALDI-TOF MS approach designed for the accurate and reliable identification of CPE including carbapenemase subtyping in Gram-negative clinical isolates. Key upgrades in A-MALDI over our previous OS-MALDI method include the optimization of MALDI-TOF settings, the application of the In-Cal process for improved mass accuracy, and the addition of an organic solvent step in sequential OS sample preparation for a target of NDM (Fig. S1). More information is described in the Supplemental Material.

### Distributions of bacterial species and carbapenemases in clinical isolates

The 469 CPE isolates included 322 *K*. *pneumoniae*, 92 *E. coli*, 27 *Citrobacter koseri*, 7 *Klebsiella aerogenes*, 7 *Klebsiella variicola*, 6 *Enterobacter cloacae*, 3 *Citrobacter freundii*, 3 *Klebsiella oxytoca*, 1 *Morganella morganii*, and 1 *Enterobacter asburiae* isolate. The 112 carbapenemase-negative isolates included 58 *E. coli*, 52 *K*. *pneumoniae*, and 2 *Klebsiella variicola* isolates (Table S1). In the analysis of all isolates, carbapenemase gene subtypes were confirmed by PCR and DNA sequencing. Totally 388 isolates harbored *bla*_KPC_ genes (383 *bla*_KPC-2_ and 5 *bla*_KPC-4_); 51 isolates harbored *bla*_NDM_ genes (26 *bla*_NDM-1_, 16 *bla*_NDM-5_, and 9 *bla*_NDM-9_); 40 isolates harbored *bla*_OXA_ genes (1 *bla*_OXA-48_, 33 *bla*_OXA-181_, and 6 *bla*_OXA-232_); and 2 isolates harbored *bla*_GES_ genes (1 *bla*_GES-5_ and 1 *bla*_GES-24_). Additionally, we identified several isolates producing two distinct carbapenemases simultaneously in a single isolate. Specifically, we observed one isolate was co-producing both *bla*_KPC-2_ and *bla*_NDM-1_; five isolates were co-producing both *bla*_KPC-2_ and *bla*_NDM-5_; three isolates were co-producing both *bla*_KPC-2_ and *bla*_OXA-181_; one isolate was co-producing both *bla*_KPC-2_ and *bla*_GES-24_; and two isolates were co-producing both *bla*_NDM-5_ and *bla*_OXA-181_.

### Analytical performance test of A-MALDI

To evaluate the effectiveness of A-MALDI, an analytical performance test was conducted to verify its performance and suitability for clinical diagnostics. We had previously tested analytical performance of OS-MALDI using selected clinical isolates following Clinical and Laboratory Standards Institute guidelines (EP12-A2) (18). Similarly, A-MALDI established a similar sample set of 12 KPC-positive and 12 KPC-negative clinical isolates tested by three experimenters over 10 days (*n* = 360 for each).

**Fig 1:**
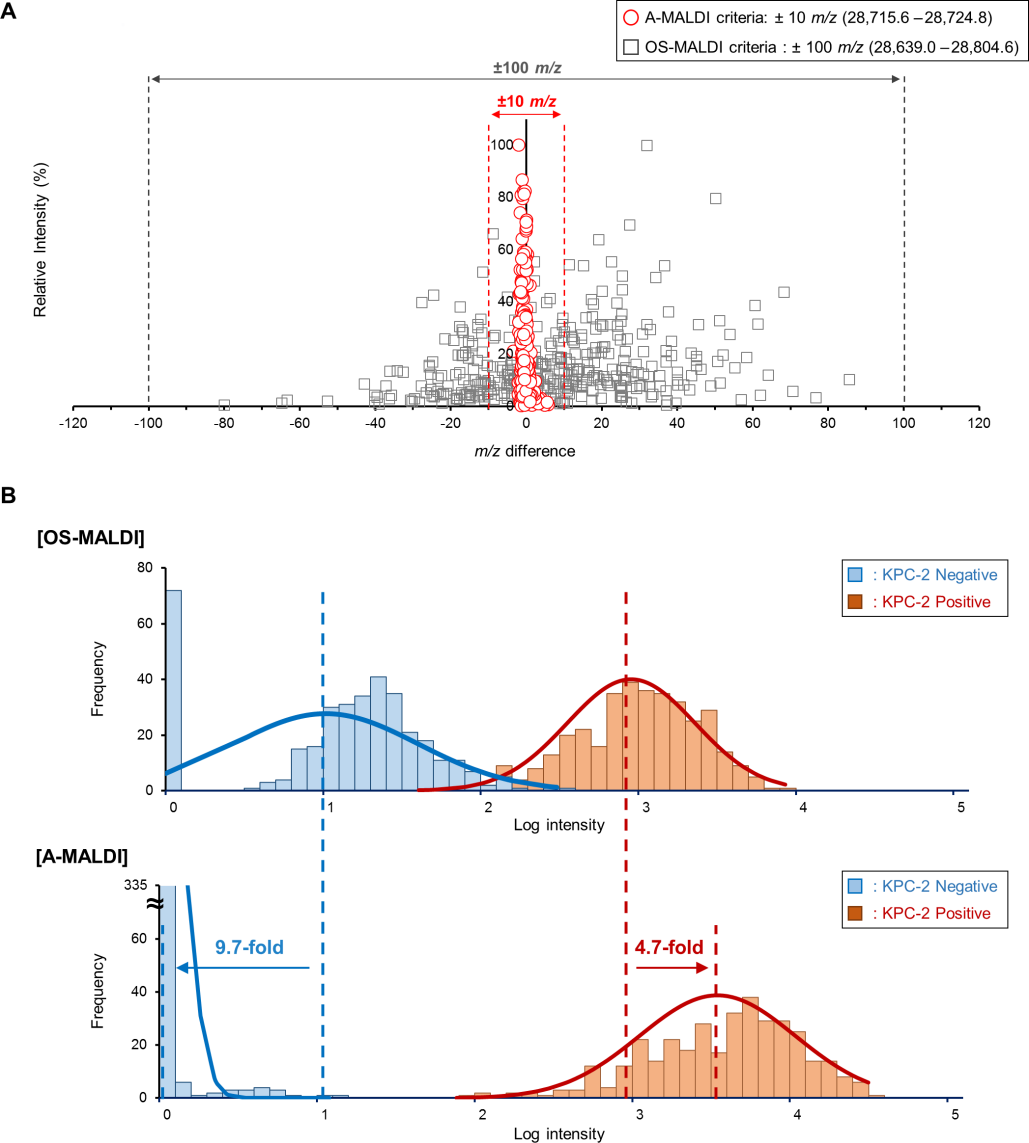
Comparison of analytical performance test for A-MALDI and OS-MALDI in KPC-2 detection. (**A**) Comparison of the mass errors in the A-MALDI (red circle), and OS-MALDI (gray squares). The scatter plot shows the mass error of each detected KPC-2 peak compared to the theoretical mass for a range of *m*/*z* values. OS-MALDI exhibits a mass distribution within ±100 *m*/*z*, whereas A-MALDI exhibits a mass distribution within ±10 *m*/*z*. (**B**) Intensity distribution of OS-MALDI (up) and A-MALDI (down). For both methods, noise signals from KPC-2-negative isolates are shown in blue, and KPC-2-positive signals from KPC-2-positive isolates are shown in red. The bar graphs represent the frequency of peak intensities picked for KPC-2, while the curved lines represent the normal distribution plots.

The results of the analytical performance test revealed that A-MALDI exhibited higher mass accuracy and detection capability than OS-MALDI in detecting KPC-2 (Fig. 1A). A-MALDI achieved a *m*/*z* distribution of 28,715.6–28,724.8, which is substantially closer to the theoretical *m*/*z* value of 28,719.11, compared to OS-MALDI’s *m*/*z* distribution of 28,639.0–28,804.6. Furthermore, the *m*/*z* variance for A-MALDI was reduced to 1.11, marking a 580-fold decrease from OS-MALDI’s variance of 581.42. This reduction in variance highlights the improved mass accuracy of A-MALDI (Table 1). Additionally, A-MALDI showed a 4.7-fold increase in KPC-2-positive signal and a 9.7-fold reduced noise in KPC-2-negative isolates compared to OS-MALDI (Fig. 1B). As a result, A-MALDI demonstrated substantially higher sensitivity and specificity in differentiating KPC-2-positive and KPC-2-negative signals compared to OS-MALDI. The representative 10-day replicate experiment for KPC-2 clinical isolate is shown in Fig. S2.

**TABLE 1:** Comparison of two analysis methods for KPC-2 identification in clinical isolates^*a*^

Samples		OS-MALDI	A-MALDI	Improvement
Analytical performance test (*n* = 12, 360 tests)	*m*/*z* distribution	28,639.0–28,804.6	28,715.6–28,724.8	Theoretical *m*/*z*: 28,719.11
	*m*/*z* variance	581.42	1.11	580-fold ↓
	Signal intensity	1,101 ± 493	5,163 ± 2,375	4.7-fold ↑
Clinical evaluation (*n* = 279^*b*^)	*m*/*z* distribution	28,632.2–28,806.6	28,714.8–28,725.6	Theoretical *m*/*z*: 28,719.11
	*m*/*z* variance	1,202.02	2.6	462-fold ↓
	Signal intensity	2,342 ± 1,362	6,462 ± 2,936	2.8-fold ↑
	True positive	268	279	+11
	False negative	11	0	−11
	Accuracy (%)	97.4	100	+2.6

^*a*^ The signal intensity shows the mean ± standard deviation.

^*b*^ The clinical isolates are overlapping samples analyzed by OS-MALDI and A-MALDI methods.

### Clinical evaluation of A-MALDI

A total of 581 clinical isolates were analyzed using A-MALDI, comprising 469 CPE isolates (388 KPC, 51 NDM, 40 OXA, and 2 GES; total of 481 CPE subtypes) and 112 carbapenemase-negative isolates. We validated the accuracy and precision of A-MALDI for identifying KPC, NDM, OXA, and GES using PCR and DNA sequencing results as references.

**Fig 2:**
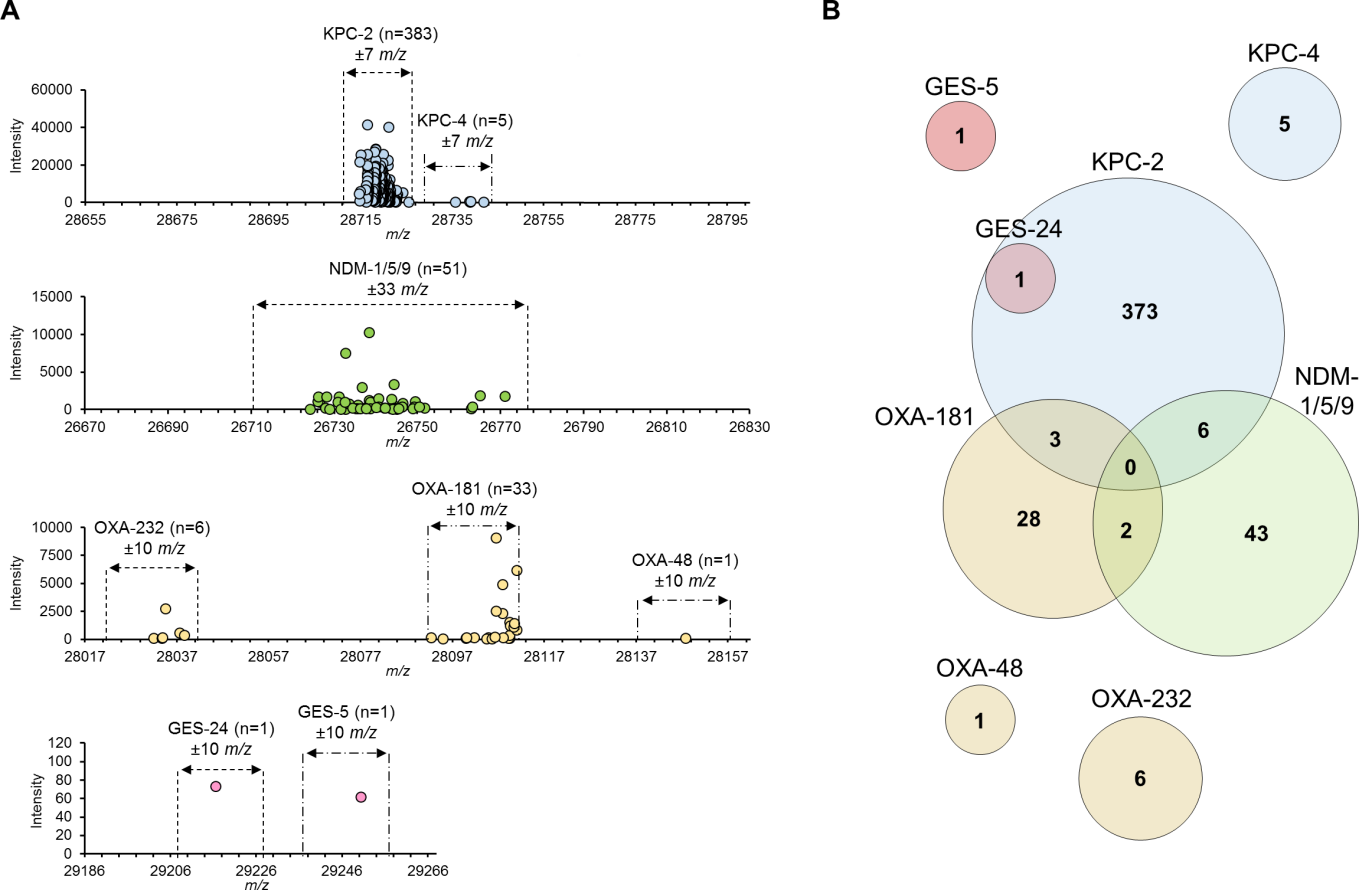
Molecular mass distribution of multiple detected CPE targets. (**A**) The graph shows the *m*/*z* distribution of the identified carbapenemase subtypes. The identification cut-off range is indicated by a dotted line based on the theoretical *m*/*z* value of the carbapenemase subtypes. (**B**) Venn diagrams showing the numbers of CPE subtypes identified by A-MALDI. Each circle represents identified CPE subtypes, with the overlapping areas of the circles indicating multiple CPE subtypes co-expressed in a single clinical isolate. Numbers in the circles indicate the number of the subtypes identified by A-MALDI to those confirmed previously by PCR and DNA sequencing.

A-MALDI demonstrated 100% accuracy and precision in identifying all collected CPE isolates (Table 2). The method accurately identified KPC, OXA, and GES carbapenemases with differences within ±10 *m*/*z*, while NDM was identified within a tolerance of ±33 *m*/*z* (Fig. 2, detailed in the Supplemental Material).

**TABLE 2:** Clinical evaluation results for the identification of multiple carbapenemases using A-MALDI

Carbapenemase	Subtypes	Identification criteria (*m/z* ± range)	Identified isolates	Accuracy% (95% CI)^*a*^*^,^*^*b*^	Precision% (95% CI)^*a*^
KPC (*n* = 388)	KPC-2 (*n* = 383)	28,719.11 ± 7	383	100 (99.3–100)	100 (99.1–100)
	KPC-4 (*n* = 5)	28,736.10 ± 7	5		
NDM (*n* = 51)	NDM-1 (*n* = 26)			100 (97.8–100)	100 (93–100)
	NDM-5 (*n* = 16)	26,743.53 ± 33	51		
	NDM-9 (*n* = 9)				
OXA (*n* = 40)	OXA-48 (*n* = 1)	28,147.68 ± 10	1	100 (97.6–100)	100 (91.2–100)
	OXA-181 (*n* = 33)	28,101.65 ± 10	33		
	OXA-232 (*n* = 6)	28,032.54 ± 10	6		
GES (*n* = 2)	GES-5 (*n* = 1)	29,247.88 ± 10	1	100 (96.8–100)	100 (15.8–100)
	GES-24 (*n* = 1)	29,217.79 ± 10	1		

^*a*^ 95% CIs were calculated using MedCalc software.

^*b*^ CI, confidence interval.

When comparing the OS-MALDI using the same 279 clinical isolates, A-MALDI showed a 2.8-fold increase (*P* < 0.001, Student’s *t*-test) in KPC-2 signal intensity. Furthermore, A-MALDI successfully identified all 11 false negatives as true positives that were missed by OS-MALDI (Table 1).

### Carbapenemase subtyping with A-MALDI

Further subtyping of CPE was feasible by discriminating KPC, OXA, and GES subtypes basically within a ±10 *m*/*z* tolerance using A-MALDI (Table 2). Specifically, our clinical isolates contained two KPC subtypes: KPC-2 and KPC-4, with a difference of 16.99 *m*/*z*. Since their identification ranges overlap by 3.01 *m*/*z*, we therefore adjusted to a slightly narrower mass range for KPC-2 or KPC-4 subtyping (±7 *m*/*z* tolerance). KPC-2 subtype (*n* = 383) was identified at 28,720.2 ± 5.4 *m*/*z* and differentiated from KPC-4 subtype (*n* = 5), having 28,738.8 ± 3.1 *m*/*z*, allowing clear discrimination (Fig. 2A). In case of one of the important KPC subtypes, KPC-3, obtained from KPC-3-producing *E. coli* standard cells, was clearly identified at 28,744.4 Da by a difference of 26 *m*/*z* from KPC-2 (Fig. S3). However, the identification range of KPC-3 overlaps with KPC-4 by 5.4 *m*/*z,* even for ±7 *m*/*z* tolerance, and it was found that KPC-3 and KPC-4 cannot be differentiated from each other by current A-MALDI. All subtypes for OXA and GES were successfully identified as follows: OXA-48 (*n* = 1) at 28,147.8 *m*/*z*, OXA-181 (*n* = 33) at 28,101.7 ± 9.4 *m*/*z*, OXA-232 (*n* = 6) at 28,035.3 ± 3.3 *m*/*z*, GES-5 (*n* = 1) at 29,250.4 *m*/*z*, and GES-24 (*n* = 1) at 29,216.5 *m*/*z*.

For NDM identification, we established a wider range of mass tolerance (26,743.53 ± 33 *m*/*z*, described in the Supplemental Material). The first reason is due to four kinds of NDM proteoforms having 14-Da mass ladder being confirmed by liquid chromatography-tandem mass spectrometry analysis and observed as a peak group in MALDI spectra (Fig. S4 to S6). The second reason is that the NDM subtypes, NDM-1/NDM-5/NDM-9, have very similar molecular weights (mass differences were each <4.01 *m*/*z*, Fig. S6B). Therefore, we identified the NDM proteins (*n* = 51) at 26,747.6 ± 23.5 *m*/*z*, applying a wider mass tolerance (±33 *m*/*z*) without subtyping discrimination in our clinical isolates (Table 2).

**Fig 3:**
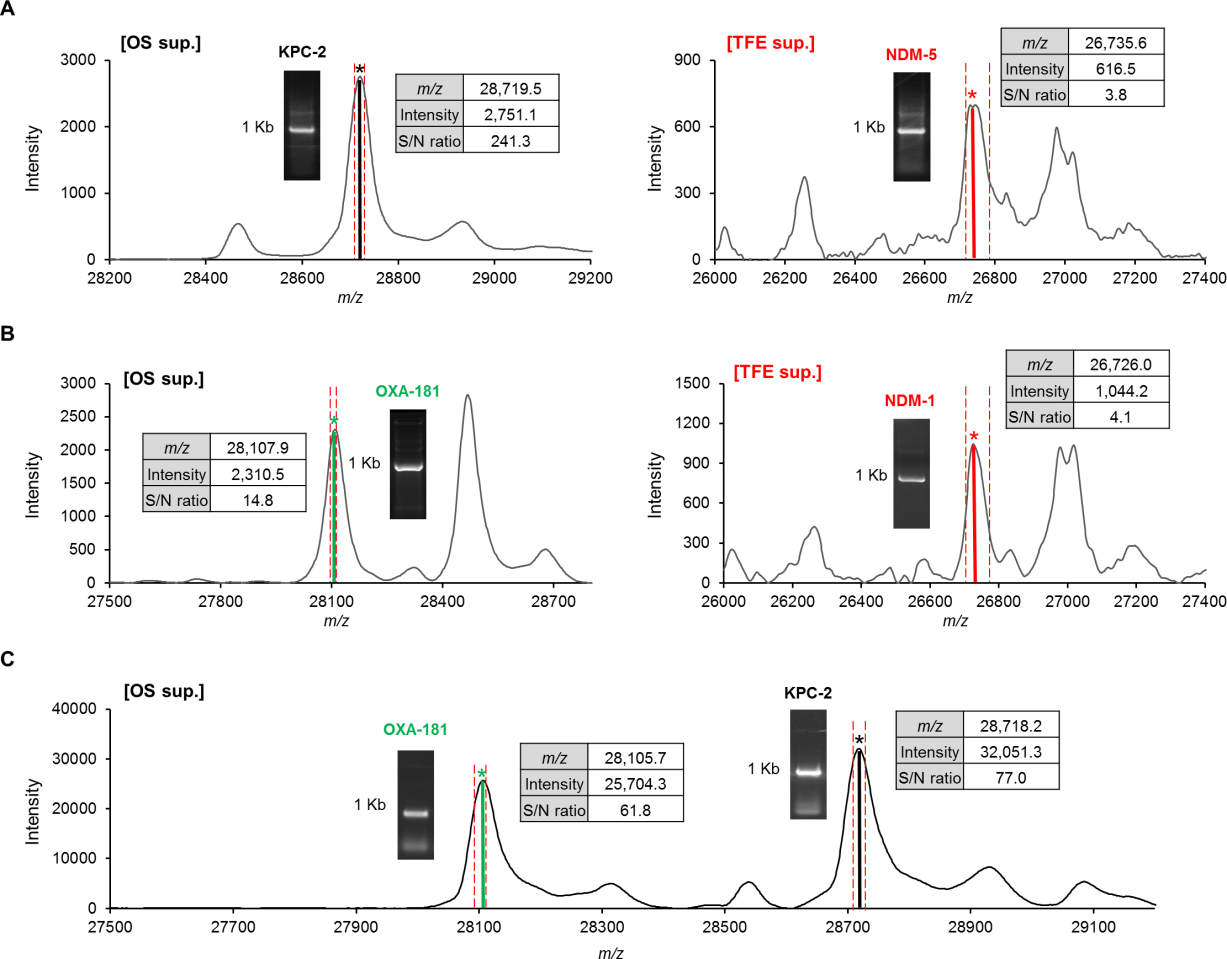
Representative MALDI-TOF MS spectra of identified co-expressed carbapenemase enzymes using the A-MALDI assay. Each panel displays a MALDI-TOF spectra of the OS or TFE supernatant of bacterial isolates co-expressing different carbapenemase enzymes: (A) KPC-2 and NDM, (B) OXA-181 and NDM, and (C) KPC-2 and OXA-181. PCR results for target proteins are included in the spectra. The red dotted line represents the identification cut-off range.

Notably, within the 469 CPE isolates, A-MALDI identified 12 co-producing strains including 6 KPC-2/NDM, 3 KPC-2/OXA-181, 1 KPC-2/GES-24, and 2 NDM/OXA-181. The co-producing strains were individually confirmed by PCR and DNA sequencing results for all four carbapenemases. Detailed sample information and the corresponding A-MALDI results are provided in Table S2, and representative MALDI spectra of these co-producing strains are shown in Fig. 3.

## DISCUSSION

A-MALDI clearly demonstrated excellent ability to identify CPEs such as KPC, NDM, OXA, and GES when carbapenemase is present in the strain (100% accuracy and precision). The method also successfully discriminated carbapenemase subtypes and simultaneous detection of co-producing multiple carbapenemases in a single strain.

Our results suggest that the A-MALDI will represent a noteworthy advancement in clinical microbiology. Previous studies have primarily focused on the direct detection of intact KPC protein (18, 21, 22), and there have been few studies targeting multi-proteins beyond KPC (e.g., NDM and OXA) using MALDI-TOF MS. One of the main reasons is that MALDI-TOF MS typically has low mass accuracy and sensitivity for high-molecular-weight protein analysis (>20 kDa) (23). Our A-MALDI demonstrated remarkable improvements compared to the OS-MALDI method. The analytical performance test results suggest its diagnostic superiority as a highly accurate and precise tool for detecting carbapenemase subtypes in 24 clinical isolates. Although our method employs the same sample lysis technique as OS-MALDI, the A-MALDI results demonstrated a significant enhancement in the intensity of the KPC-2 signal, showing a remarkable increase (*P* < 0.001), while concurrently, the *m*/*z* variance and the false positives were substantially minimized. In clinical evaluations, A-MALDI demonstrated also superior results compared to the OS-MALDI method. For KPC detection, while OS-MALDI reported 97.4% accuracy in a previous clinical evaluation, A-MALDI achieved 100% accuracy in the current study (Table 1). This improvement may be attributed to the synergistic effect of A-MALDI regarding sensitivity and accuracy increase. A-MALDI also successfully identified additional types of carbapenemases, NDM, OXA, and GES, with 100% accuracy and precision (Table 2). There was no exception for bacterial species (total 10 species) in the CPE identification and subtyping (Table S1). To the best of our knowledge, this is the first report for simultaneous and multiple detection of intact carbapenemases of KPC, NDM, OXA, and GES in a clinical isolate.

Clinical needs for CPE subtyping are increasing due to emerging threats from carbapenemase mutants, which result in treatment failure (13). Here, we address the accurate subtyping for CPEs. In fact, more than 150 *bla*_KPC_ variants have been reported worldwide, and most of the new variants were discovered in the past 3 years, which calls for public alarm (13). Currently, the most frequently found KPC subtypes in South Korea are KPC-2, KPC-3, and KPC-4. A-MALDI can successfully identify KPC and also discriminate KPC-2 with KPC-3/KPC-4.

The detection of OXA-48-like carbapenemases presents specific challenges in detection due to their relatively weak carbapenemase activity, making it challenging for certain phenotypic methods to accurately detect them (24). However, in our study, OXA carbapenemases, specifically OXA-48, OXA-181, and OXA-232, were accurately identified, with OXA-181 being the most frequently detected subtype (*n* = 33). OXA-181 is the second most common OXA-48-like subtype globally (24). Additionally, co-expressing strains, such as NDM-5 with OXA-181, have been reported in Korea, further complicating treatment options and highlighting the importance of precise detection strategies (25). While GES subtypes are present at a low prevalence in Korea, we were successfully able to identify a single isolate for each GES (GES-5 and GES-24) subtype.

Since NDM is known as a membrane-anchored protein (26), it was not detected by the OS lysis method. In fact, the detection of NDM proteins in MALDI-TOF MS analysis is very challenging since NDM has a membrane-like molecular property due to lipidation on the cysteine residue (26), making it prone to loss in sample preparation steps prior to analysis. Therefore, we additionally developed an orthogonal method to extract NDM protein from membrane compartments using the organic solvent (i.e., TFE). We next used an incorporated ion exchange resin and non-ionic detergent into the organic solvent-based lysis method, which significantly improved detection sensitivity and accuracy. Unlike KPC, OXA, and GES, the NDM-1 subtype has similar molecular masses with other subtypes, NDM-5 or NDM-9, which are not distinguishable within ±10 *m*/*z* tolerance. Moreover, we found that NDM-1 also has three other proteoforms with 14-Da differences in *E. coli* (Fig. S5). To address this issue, we set a relatively wider mass tolerance (±33 *m*/*z*).

Importantly, the clinical isolates applied in this study (~98.8%) mostly covers the CPE subtypes, which are highly frequent and clinically relevant in hospitals in South Korea. We first started to focus on two subtypes of KPC (KPC-2 and KPC-3/KPC-4), three subtypes of OXA (OXA-48, OXA-181, and OXA-232), and two subtypes of GES (GES-5 and GES-24) from all the CPE isolates for A-MALDI evaluation. Accurate identification and distinction of carbapenemase co-producing strains in such clinical isolates will be crucial for optimal patient management and treatment selection (27).

However, our current study has several limitations. First, we were unable to collect samples harboring VIM and IMP yet. Additionally, we were able to analyze only a relatively small number of samples for specific subtypes, such as OXA-181, GES-5, and GES-24, due to the CPE prevalence distribution in South Korea: KPC (81.2%), NDM (13.8%), OXA (3.7%), IMP (0.1%), VIM (0.1%), and GES (< 0.1%). Second, MALDI-TOF analysis requires bacterial colonies obtained from cultured samples, necessitating a time-consuming bacterial culture step before analysis. Lastly, our A-MALDI method is limited to detecting only CPE strains in CRE isolates. The inability to detect non-CPE isolates (<6%) represents a limitation in the comprehensive surveillance of carbapenem resistance. Non-CPE isolates were not assessed in this study, and this potential was not validated in the current study. Further studies are needed to develop complementary techniques, providing a more complete identification of CRE in clinical settings.

Carbapenemase genes, such as *bla*_KPC_, can encode multiple enzyme subtypes with differing substrate specificities and resistance levels. For example, KPC-2, KPC-4, and KPC-5 are subtypes of the KPC enzyme that differ by only a few amino acids, yet they can have significantly different hydrolytic activities and kinetic properties of the enzymes against carbapenem antibiotics (28). Accurate identification of specific carbapenemase subtypes is clinically relevant, as different enzymes exhibit varying levels of resistance to carbapenem antibiotics (29–33). This variability emphasizes the importance of customizing antimicrobial therapy based on the specific carbapenemase subtype identified, as different subtypes may demonstrate variable susceptibility profiles to available treatment options. To further improve clinical outcomes, incorporating the pharmacokinetic/pharmacodynamic index (34) for individual subtypes with our A-MALDI results might aid in making informed recommendations for antibiotic therapies. In addition, A-MALDI’s ability to provide detailed identification of carbapenemase subtypes significantly may enhance epidemiologic surveillance efficiency, specially for LTCFs. By accurately identifying and tracking the prevalence and spread of specific carbapenemase-producing strains, A-MALDI is expected to support public health initiatives aimed at controlling antibiotic resistance. Furthermore, A-MALDI’s rapid identification capabilities offer significant advantages in clinical settings by providing faster results after bacterial culture compared to traditional methods (Fig. S7). For A-MALDI, the process begins with the collection of colonies following 16–20 h of bacterial culture, a step that takes only a few seconds. This is followed by four sequential buffer incubations (total of 40 min, 10 min/step) and each centrifugation in between incubation (total of 30 min, 10 min/step). The final step, MALDI-TOF MS analysis, requires approximately 5 min. In total, the A-MALDI process, from colony collection to final analysis, takes approximately 1 h and 15 min (Fig. S1 and S7). As carbapenem-resistant superbugs continue to rise, the development of new therapeutic options will become increasingly important (35). Our study expects to contribute by enabling the timely detection of antibiotic-resistant subtypes, potentially informing and guiding the development of future treatments.

In conclusion, this study demonstrates the potential of A-MALDI to enhance diagnostic accuracy, guide patient treatment decisions, and improve infection control through precise identification of carbapenemase subtypes.

## Data Availability

All experimental data using R packages have been deposited to the ProteomeXchange Consortium via the PRIDE partner repository with the dataset identifier PXD058284.

## SUPPLEMENTAL MATERIAL

Supplemental material jcm.01475-24-s0001.docx Supplemental methods and results, Figures S1 to S8, and Table S1.

Table S2 jcm.01475-24-s0002.xlsx Clinical test results of an A-MALDI in 581 clinical isolates.

ASM does not own the copyrights to Supplemental Material that may be linked to, or accessed through, an article. The authors have granted ASM a non-exclusive, world-wide license to publish the Supplemental Material files. Please contact the corresponding author directly for reuse.
