# Supplementary material for: Clinical evaluation of advanced MALDI-TOF MS for carbapenemase subtyping in Gram-negative isolates
Source: J Clin Microbiol. 2024 Nov 29;63(1):e01475-24. doi: 10.1128/jcm.01475-24 (PMC11784181; doi:10.1128/jcm.01475-24)
Supplement: Supplemental material — Supplemental methods and results, Figures S1 to S8, and Table S1. [file jcm.01475-24-s0001.docx]

**Clinical Evaluation of Advanced MALDI-TOF MS for Carbapenemase Subtyping in Gram-negative Isolates**

Dong Huey Cheon^1^, Heejung Jang^1^, Yoon Kyung Choi^1^, Won Seok Oh^1^, Seohyun Hwang^1^, Ju-Ri Park^1^, Hyojin Kim^1^, Yoonha Park^1^, Saeyoung Lee^1^, Won Suk Yang^1^, Min Jin Kim^2^, Sun Hwa Lee^2^, and Je-Hyun Baek^1*^

^1^R&D Center for Clinical Mass Spectrometry, Seegene Medical Foundation, Seongdong-gu, Seoul, Republic of Korea. ^2^Department of Laboratory Medicine, Seegene Medical Foundation, Seongdong-gu, Seoul, Republic of Korea.

*Corresponding author:

E-mail: jhbaek@mf.seegene.com

**Supplementary Methods**

**Identification of microorganisms using MALDI-TOF MS**

The bacterial identification of clinical isolates using MALDI-TOF MS was performed by routine clinical assay (1). MALDI-TOF-MS measurement was performed using the MALDI Biotyper Compass Explorer version 4.1 and the Microflex LT/SH smart MALDI-TOF MS system (Bruker Daltonics GmbH & Co. KG, Germany), which allows for the identification of microbial species. The MBT Compass reference library covers 3,893 species from 664 genera of microorganisms, and the identification threshold is set at 2.0.

**Detection of carbapenemase genes using target gene-specific primers**

Carbapenemase genes were detected by PCR using primers designed to capture each corresponding target DNA. PCR amplification was performed using the CFX96 Real-Time System (Bio-Rad Laboratories, Inc., USA). The *bla*_KPC-2_ and *bla*_KPC-4_ genes, encoding 293 amino acids, were amplified using the forward primer 5ʹ-ATGTCACTGTATCGCCGTCTAG-3ʹ and the reverse primer 5ʹ-TTACTGCCCGTTGACGCCC-3ʹ. The *bla*_NDM-1_, *bla*_NDM-5_, and *bla*_NDM-9_ genes, encoding 270 amino acids, was amplified using the forward primer 5ʹ- ATGGAATTGCCCAATATTATGCAC-3ʹ and the reverse primer 5ʹ- TCAGCGCAGCTTGTCGGC-3ʹ. The *bla*_OXA-48_ gene, encoding 265 amino acids, was amplified using the forward primer 5ʹ-ATGCGTGTATTAGCCTTATCGG-3ʹ and the reverse primer 5ʹ-CTAGGGAATAATTTTTTCCTGTTTGA-3ʹ. The *bla*_OXA-181_ and *bla*_OXA-232_ genes, encoding 265 amino acids, were amplified using the forward primer 5ʹ-ATGCGTGTATTAGCCTTATCGG-3ʹ and the reverse primer 5ʹ-CTAGGGAATAATTTTCTCCTGTTTG-3ʹ. The *bla*_GES-5_ and *bla*_GES-24_ genes, encoding 287 amino acids, were amplified using the forward primer 5ʹ- ATGCGCTTCATTCACGCACTAT -3ʹ and the reverse primer 5ʹ-CTATTTGTCCGTGCTCAGGAT-3ʹ. All PCR products were resolved by 0.8% agarose gel electrophoresis and confirmed by DNA sequencing (Cosmogenetech Inc., Republic of Korea).

**Purification of KPC-2 standard proteins and preparation of standards**

Recombinant KPC-2–producing *E. coli* standard cells were produced as previously described (2). Ion exchange chromatography was performed according to the manufacturer's instructions, with slight modifications. Following the manufacturer's protocol, the FT fraction containing KPC-2 protein was subjected to sample enrichment and buffer exchange using an Amicon Ultra-15 10k Centrifugal Filter (MilliporeSigma, USA). The final concentration of purified KPC-2 protein was determined by a NanoDrop spectrophotometer (Thermo Fisher Scientific Inc., USA).

As a negative control to serve as a CPE-negative matrix, we utilized osmotic shock supernatant (OS sup) of the *E. coli* TOP10 competent cells (Yeastern Biotech, Taiwan). The growth of *E. coli* cells was controlled by the culture broth (Luria-Bertani), incubation time (16–20 h), optical density (OD600 of 2.1 to 2.4), and the protein concentration of the OS lysate (0.15–0.2 mg/mL). Finally, well-controlled standards were prepared by spiking purified KPC-2 protein onto the CPE-negative matrix.

**Preparation of NDM-1–producing *E. coli* standard cells**

The NDM-1–producing *E. coli* standard cells were prepared as follows. The *bla*_NDM-1_ gene was amplified by PCR using the following primers: forward primer, 5’- AACTGCAGGATGGAATTGCCCAATATTATGCA-3’ (32-mer); and reverse primer, 5’- GGAATTCTCAGCGCAGCTTGTCGG-3’ (24-mer). The size of the PCR products was confirmed by 0.8% agarose gel electrophoresis, and the presence of the *bla*_NDM-1_ gene was verified through DNA sequencing analysis performed by Cosmo Genetech (Republic of Korea). Subsequently, the PCR products were digested with PstI and EcoRI and cloned into the pUC19 vector. Finally, the resulting cloned vector containing the NDM-1 gene was transformed into competent *E. coli* TOP10 cells obtained from Yeastern Biotech (Taiwan).

**Bottom-up analysis of NDM-1 using a Q-Exactive HF-X mass spectrometer**

NDM-1-producing *E. coli* standard cells were cultured in LB broth containing 100 μg/mL ampicillin and 100 μM ZnSO_4_ at 37°C for 16 h. Bacterial cells were harvested by centrifugation at 14,000 × *g* for 10 min at 4°C. After OS lysis, the resulting cell pellets were resuspended in 100 μL of 10–90% TFE. The resuspended cells were incubated and centrifuged at 14,000 × *g* for 10 min at RT. The supernatant was collected, and 15 μL of the sample was loaded onto a 15% SDS-PAGE gel. The NDM-1 proteins were visualized using the Coomassie staining method**.** The NDM-1 protein was excised from the gel lane corresponding to the 20% TFE fraction, followed by in-gel digestion (3) and LC-MS/MS analysis using a Thermo Q-Exactive HF-X mass spectrometer (2), as reported previously.

**Intact protein analysis of NDM-1 using a Q-Exactive HF-X mass spectrometer**

NDM-1–producing *E. coli* standard cells were harvested by centrifugation at 14,000 × *g* for 10 min at 4°C. Cells were then resuspended in 50 mM ammonium bicarbonate buffer and lysed by sonication (40 Hz, 200 W) in 10 mM phenylmethylsulfonyl fluoride for 5 min. After centrifugation at 14,000 × *g* for 10 min at 4°C, the supernatant was reduced by 50 mM DTT for 10 min at 37°C. The protein sample including NDM proteoforms was prepared by the following protein precipitation method (4). Briefly, to precipitate proteins, we used a volume ratio of 1:1:4:3 of protein sample:chloroform:methanol:water. The supernatant was removed by aspiration, and the precipitated pellet was further washed with one more round of addition and removal of methanol. Pellets were re-solubilized for 15 min at -20°C using 5 μL of 80% formic acid and then diluted with 95 μL water (final 4% formic acid). The sample was immediately analyzed on an LC-MS/MS system coupled with an in-house PLRP-S column (length: 10 cm; ID: 10 μm; pore size: 4,000 Å), Ultimate 3000 UPLC system, and QE-HFX mass spectrometer. An 81 ng protein sample was injected by the autosampler. The flow rate was set to 1.5 μL/min, and the gradient condition was set to 5% B for 5 min, 5–10% for 0.1 min, 10–70% for 15.4 min, 70–95% for 0.5 min, 95% for 0.5 min, 95–5% for 0.5 min, and 5% for 8 min (total run time: 30 min). MS acquisition was performed with the following parameters: 900–1,250 *m/z* scan range, 2 microscan, 120,000 resolution, and 1,500 msec max ion time.

**Deconvolution of mass spectra and conversion to average mass**

Merged mass spectra for NDM-1 charged clusters at 19–20 min retention time range were deconvoluted using the Xtract algorithm of the FreeStyle Software (Thermo Fisher). Deconvolution parameters were as follows. Data selection was performed with 900–1,250 *m/z* range in the spectrum. Adduct element was set to proton mass (H^+^: 1.00727663 Da). Charge range was set to 20–30. Analyzer type was set to OT. Minimum number of detected charge states was set to three. Relative abundance threshold (%) was set to 20. Deconvoluted masses (monoisotopic masses) were converted to average mass for MALDI-TOF MS analysis. Based on an averagine model (^12^C: 4.9384, ^1.0078^H: 7.7583, ^15.9949^O: 1.4773, ^14.0031^N: 1.3577, ^31.9721^S: 0.0417), the mass portion of each atom that contributed to the monoisotopic mass of the NDM-1 proteoform was calculated. Then, the portion was multiplied by the average mass of each atom (5). Finally, the sum of all the results was assumed as the average mass of each proteoform.

**Supplementary Results and Discussion**

**Extraction of NDM protein using TFE organic solvent**

NDM protein has been known as a protein also having a lipid and regulating antibiotics resistance as locating at bacterial membrane (6). We obtained an organic-favorable fraction using a TFE and evaluated the extraction efficiency of NDM protein for a membrane fraction from NDM-1–producing *E. coli* standard cells. Our results indicated that the highest extraction efficiency for NDM protein was achieved within the range of 20–40% TFE concentration (**Fig. S4A**). Through the optimal TFE lysis method, we effectively extracted the NDM-1 protein from NDM-producing bacterial cells and confirmed the NDM protein in the MALDI-TOF MS analysis. The NDM-1 protein was detected mainly at 26,738.4 m/z by A-MALDI (asterisk). Interestingly, two or three of additional NDM-specific peaks were also detected around the main peak (**Fig. S4C**).

**Identification of NDM-1 proteoforms by LC-MS/MS**

The NDM-1 protein was identified using in-gel digestion of TFE lysate and the nanoflow-LC(C18)-MS/MS system (**Fig. S4B**). The results revealed 73% coverage of the NDM-1 protein sequence. Lipidated or non-lipidated proteoforms of the NDM-1 protein were not distinguishable in this bottom-up analysis. However, in the intact protein analysis, we observed four major proteoforms of the NDM-1 protein (**Fig. S5**). Interestingly, each extracted ion chromatogram (XIC) of the four proteoforms (❶ 990.17 *m/z*, ❷ 991.29 *m/z*, ❸ 991.81 *m/z*, and ❹ 992.33 *m/z* at a retention time of 19~20 min) was distinguishable as an individual chromatogram with exactly 0.52 *m/z* difference at +27 charge state (this indicates 14.02 Da mass distance), which showed different retention times of 0.22~0.34 min. The multiple charged envelopes showed very similar patterns and the same charge states within the corresponding charged clusters at 19~20 min retention time (**Fig. S5B–E**). Through deconvolution of the spectra, we obtained the monoisotopic masses for the proteoforms from the mass spectra (**Fig. S5D**). Each XIC corresponds to ❶ 26,707.6557 Da, ❷ 26721.6487 Da, ❸ 26,735.6749 Da, and ❹ 26,749.7592 *m/z*, respectively, as monoisotopic masses. The monoisotopic masses were congruent with the 14.02 Da mass distance in the calculation of the precursor masses, which may imply the addition(s) of -CH2- to the lipid branches on the first proteoform (❶). We suggest that the difference in retention time due to the addition of methyl groups may have influenced the right shift of the retention time in the LC-MS/MS analysis, reflecting an increase in hydrophobicity. Such a pattern has been often observed in the variation of lipid chains (7). Furthermore, we speculate that the different changes in retention times (0.34 min for ❶-❷, 0.22 min for ❷-❸, and 0.22 min for ❸-❹) are due to the location of the methyl groups on different lipid branches (**Fig. S5B and E**).

Our A-MALDI approach is based on MALDI-TOF data, which show the average mass of protein targets. Therefore, we need the average mass values that can be obtained from the identification of NDM proteoforms with high-resolution mass spectrometry. For this, we converted the monoisotopic masses of the NDM proteoforms to average masses using essential information, such as each monoisotopic mass and averagine mass, obtained with the isotope distribution calculator (IDCalc) generated from MacCoss Lab at the University of Washington (5). The converted average masses of the NDM proteoforms were ❶ 26,724.53 Da, ❷ 26,738.53 Da, ❸ 26,752.53 Da, and ❹ 26,766.53 Da. We confirmed that these were well-detectable in MALDI-TOF mass spectra within ±10 *m/z* (**Table S2**). When considering a mass error of ±10 *m/z*, because the mass ranges for each identifiable NDM proteoform overlapped by as much as 3 *m/z*, the total range for NDM-1 identification became 26,714.53–26,776.53 *m/z* (26,745.53 ±31 *m/z*). When considering the mass difference of -4.01 Da between NDM-1 and NDM-5, and accounting for the smaller difference of -1.19 Da between NDM-1 and NDM-9, the total range for NDM (NDM-1, NDM-5, or NDM-9) identification became slightly wider at 26,710.52–26,776.54 *m/z* (26,743.53 ±33 *m/z,* **Fig. S6**).

For NDM identification, we selected peaks from the A-MALDI spectra that met the mass value criteria and had a signal-to-noise ratio greater than 1.5. These peaks were then considered as correct identifications.

**Matrix adduct peak exclusion**

One challenging issue in our MALDI-TOF MS analysis was the presence of matrix adduct ion peaks resulting from the use of SA. These adduct peaks were observed at roughly +208 *m/z* due to adducts of proteins (8) with relatively high intensities. The SA adduct peaks increased false-positive rates for target proteins (e.g., KPC-4) adjacent to a strong protein peak commonly observed in *K. pneumoniae* (28,539 *m/z*). Therefore, we performed an accumulative peak-pattern analysis of target protein peaks and adjacent SA adduct peaks using our in-house R package. We extracted the SA adduct peaks from all KPC-2 data (360 tests) acquired in the analytical performance test. We then compared the relative intensities and the *m/z* distances between the KPC-2 peaks and their adduct peaks (**Fig. S8 A**; Note that there is no protein at the *m/z* value of the SA adduct in the negative test set). The mass distance between KPC-2 and the observed SA adducts ranged from 182.9 *m/z* to 233.2 *m/z*. Based on this result, we set the SA adduct peak recognition range to 180 *m/z* to 235 *m/z*. The relative intensity (%) of the adduct peak did not exceed 41.5% of the target peak intensity. **Figs. S8 B**–**D** shows a representative spectrum for distinguishing between a SA adduct peak and KPC-4 in clinical isolates. Both peaks are found following a 28.5 kDa protein peak and within the identification cut-off range for KPC-4 protein. The relative intensity of the KPC-4 peak against the 28.5 kDa peak has a minimum value of 48.7%. Therefore, the cut-off threshold for recognizing the adduct peak is established based on a relative intensity of 45% within the 180–235 *m/z* range. If the relative intensity of the following peak is over 45%, the following peak is recognized as a protein peak. Otherwise, it is recognized as a SA adduct peak. All five KPC-4 proteins were identified among the 469 CPE clinical isolates when the adduct peak cut-off was applied, with no false positives. When no adduct peak cut-off was applied, eight false positives occurred among 112 non-CP-CRE clinical isolates, resulting in a 7% decrease in specificity and accuracy.


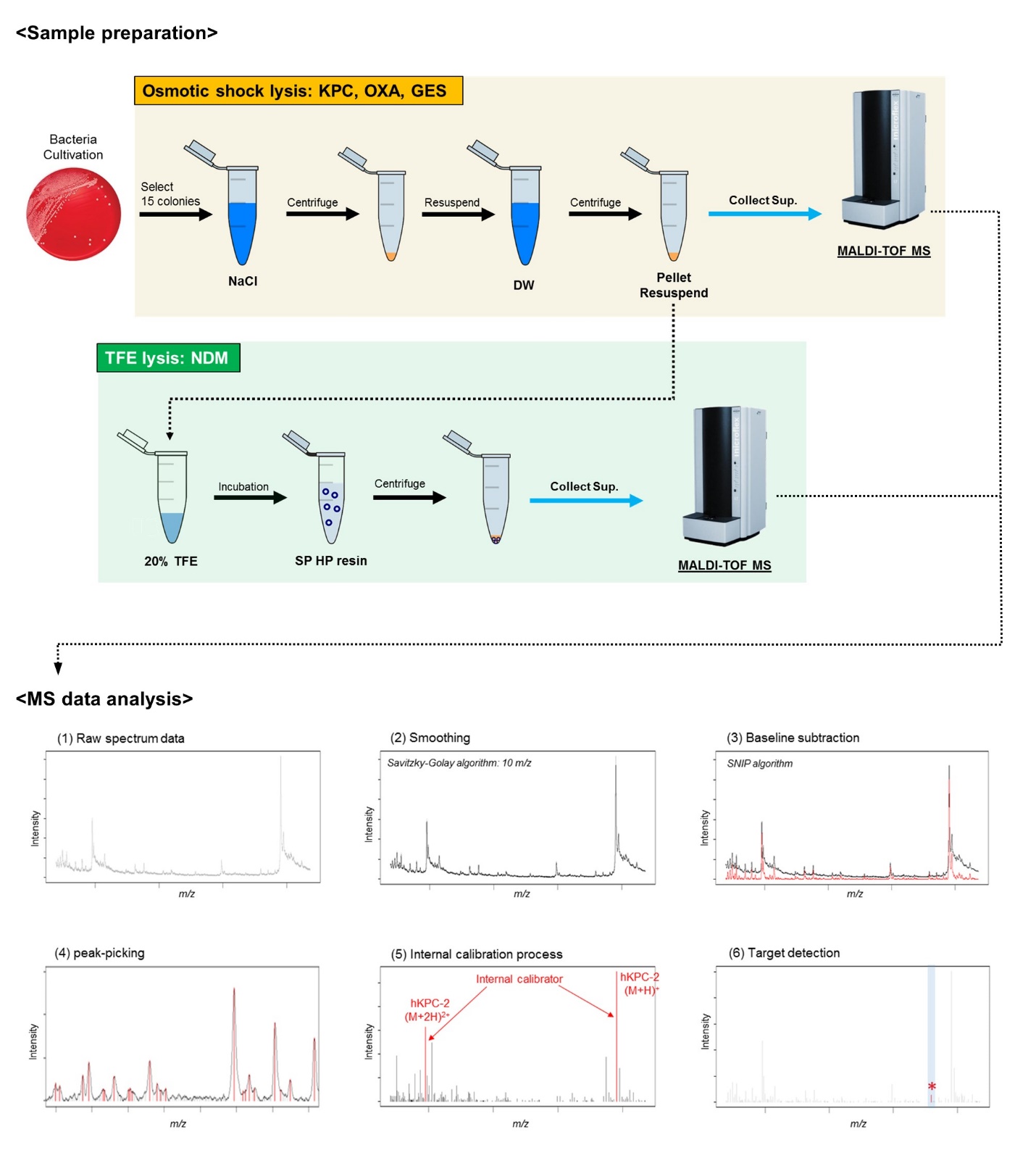


**Fig. S1.** **Workflow of A-MALDI method for CPE Identification**

(Full spectrum)


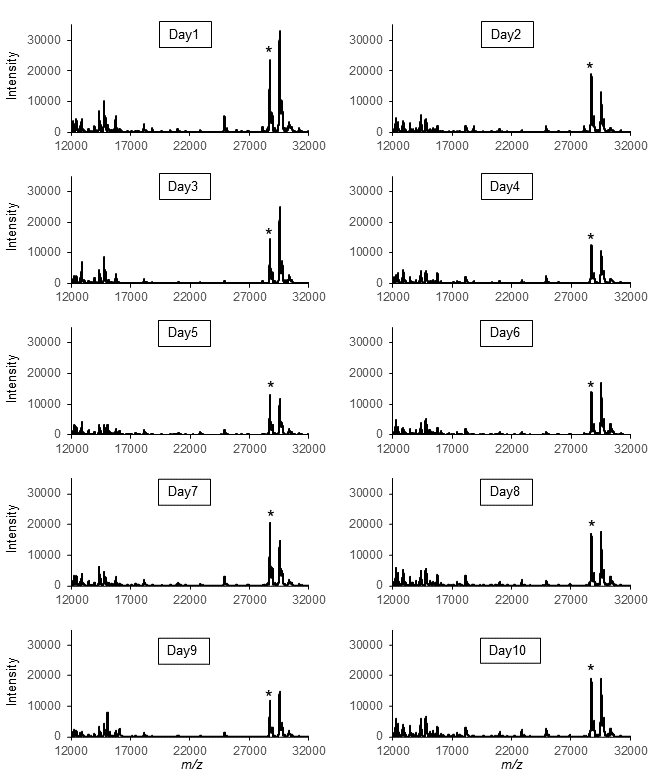


(continued, zoomed)


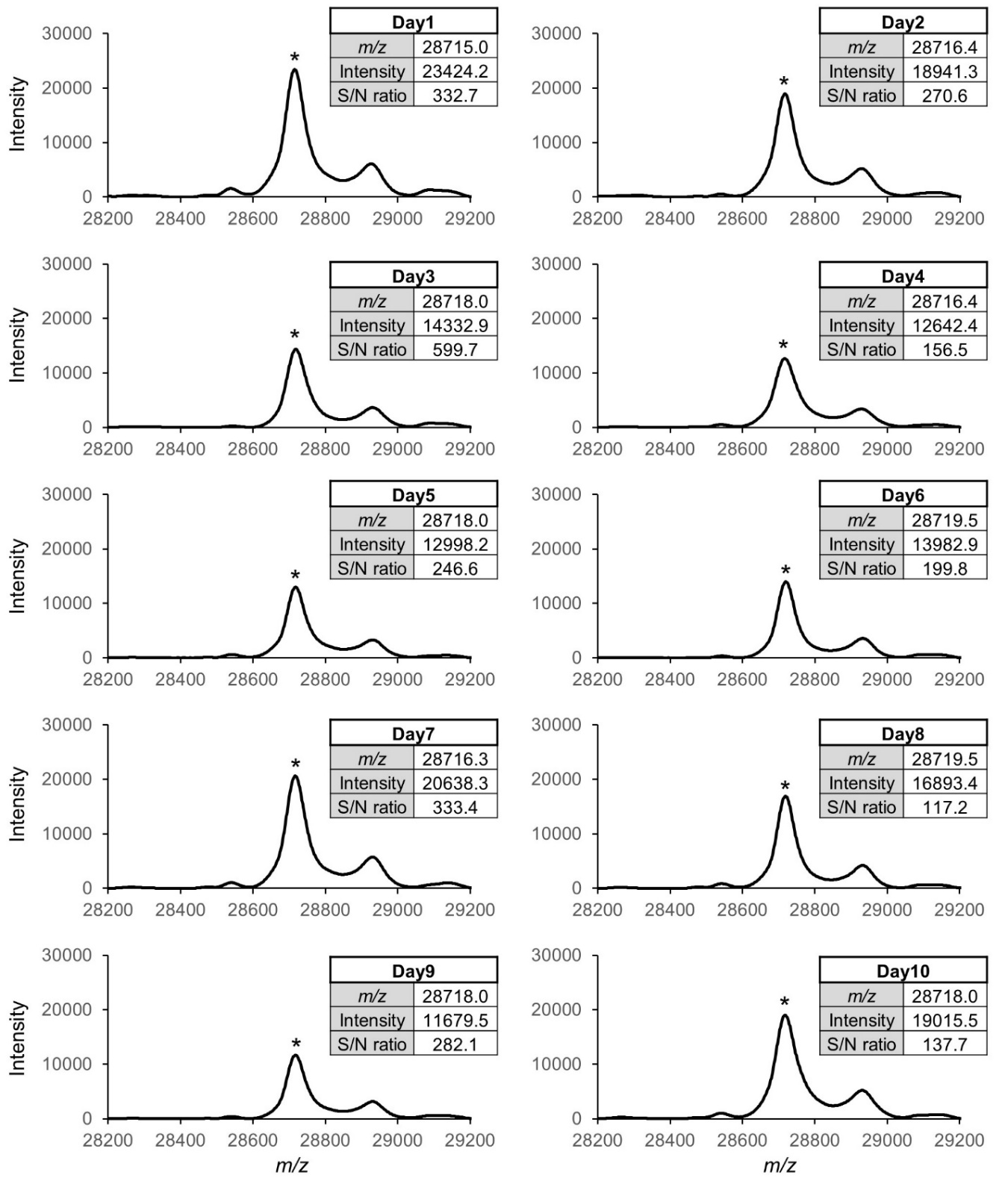


**Fig. S2. Representative replicate data over 10 days for KPC-2 clinical isolates using A-MALDI**

The upper figure displays the full MALDI-TOF MS spectrum obtained across 10 consecutive days. The lower figure provides a zoomed-in view of the region where the KPC-2 protein peak is detected. The asterisk (*) indicates the position of the KPC-2 peak. An internal calibration process was conducted for each experiment to ensure accuracy.


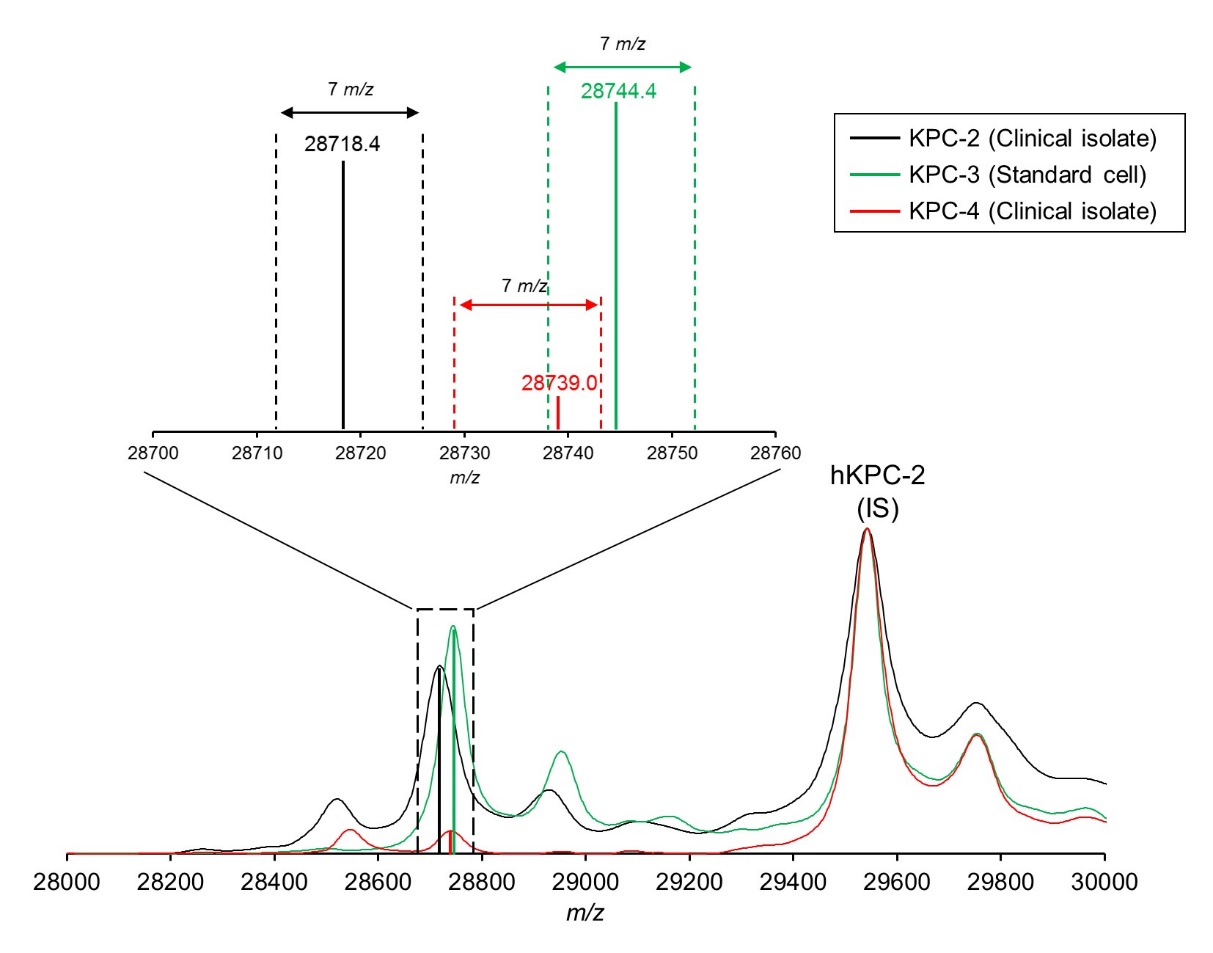


**Fig. S3.** **Discrimination of KPC subtypes (KPC-2, KPC-3, and KPC-4) in A-MALDI**

All MALDI spectra were internally calibrated with hKPC-2 IS. Three spectra were merged in a graph at the bottom. Zoomed region with dashed box was highlighted as centroid spectra at the top. KPC-2 (black color) and KPC-4 (red color) data were representatively selected from clinical isolates, while KPC-3 (green color) data was obtained from KPC-3-producing standard *E.coli* cells. The *m/z* range for the KPC subtypes identification was indicated with dashed lines (within ±7 *m/z*).


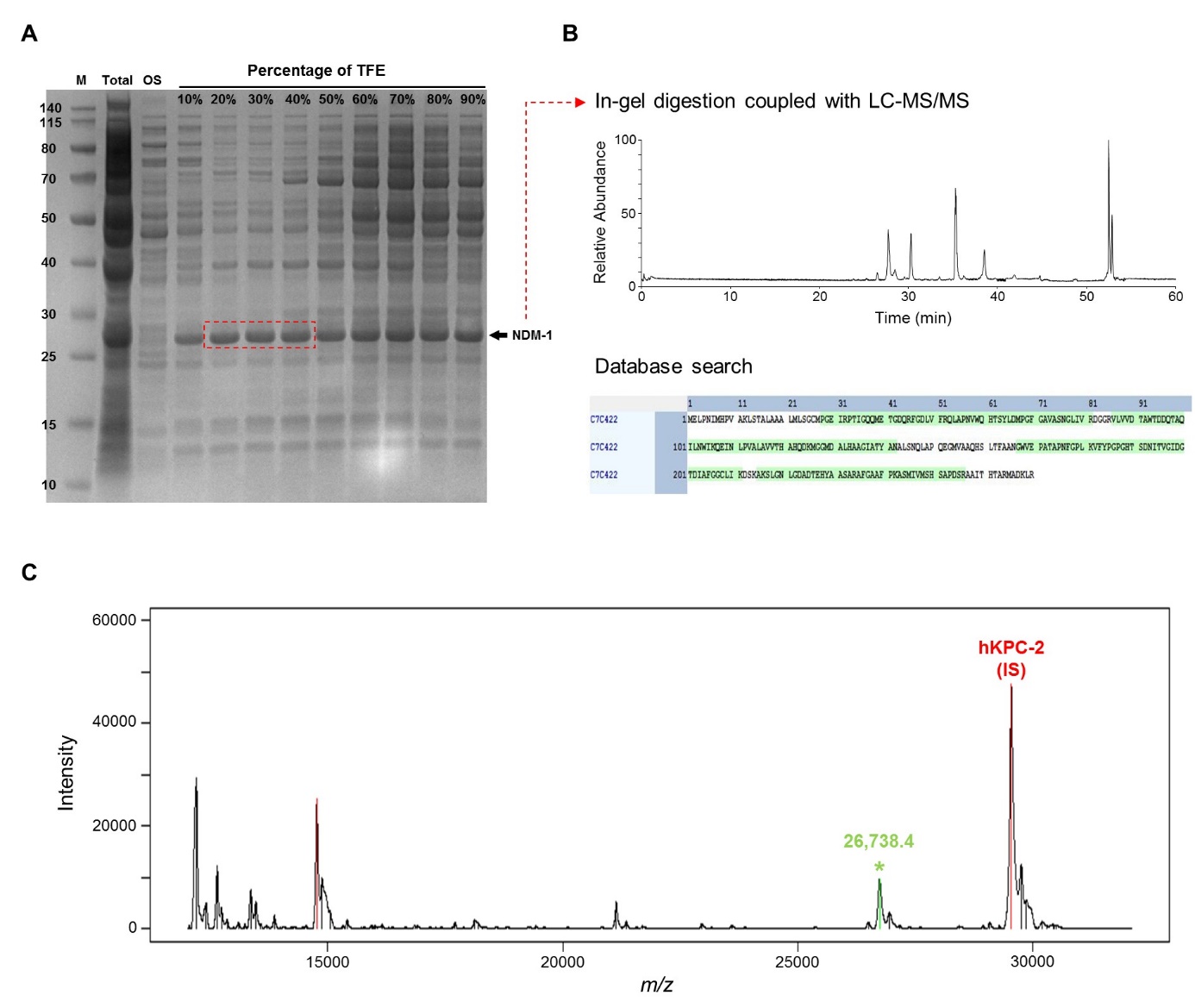


**Fig S4. MALDI-TOF MS and LC-MS/MS results for NDM-1 protein**

**A,** SDS-PAGE image of NDM-1 protein extracted by TFE lysis. The supernatant fraction shows the effect of NDM-1 protein extraction with each concentration of TFE. The portion indicated by the black arrow corresponds to the molecular weight of NDM-1. The red dotted box indicates the TFE concentration that shows the optimal efficiency of NDM-1 elution. **B,** Simplified illustration of the in-gel digestion for identifying NDM-1 protein in the TFE lysate. **C,** MALDI-TOF results of NDM-1 protein detected in TFE lysate. The green line represents the peak of the NDM-1 protein (asterisk), while the red line represents the internal standard.


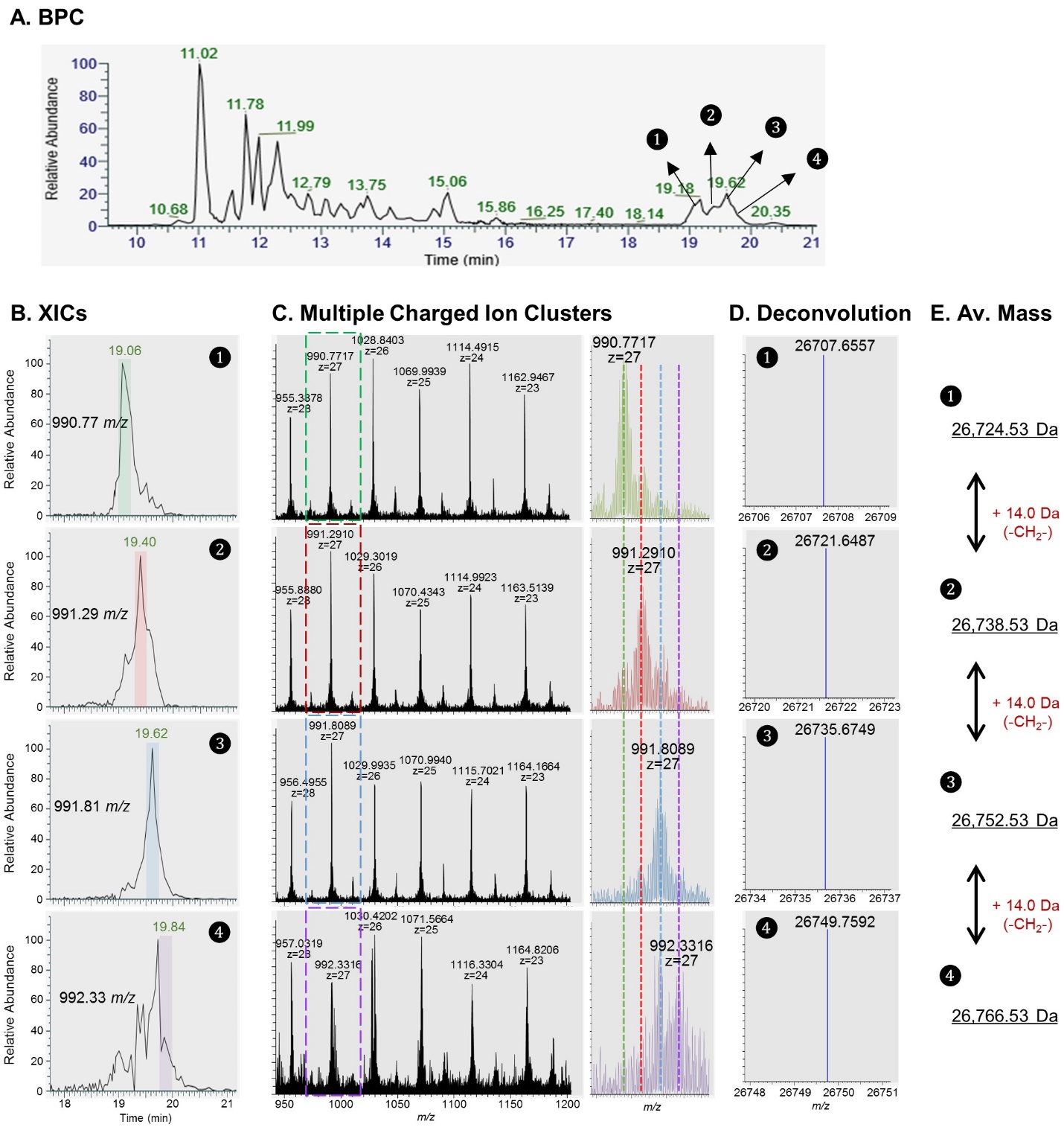


**Fig. S5. Determination of the average mass of four NDM-1 proteoforms**

**A,** Base peak chromatogram (BPC) for a proteome fraction from NDM-1-producing *E. coli* cells. Arrows represent the elution profile of four NDM-1 proteoforms. **B,** Extracted ion chromatograms (XICs) for the four NDM-1 proteoforms (❶ 990.77 *m/z*, ❷ 991.29 *m/z*, ❸ 991.81 *m/z*, ❹ 992.37 *m/z*). Isolation width for the XICs was set to 5 ppm. **C,** Multiple charged ion clusters for each NDM proteoform (left) and zoomed images of selected ion clusters (colored dotted boxes) with +27 charge states (right). Dashed lines on the left indicate the center masses of the four ion clusters. **D,** Calculated deconvoluted monoisotopic masses of the NDM-1 proteoforms. **E,** converted average masses showing +14 Da difference.

**
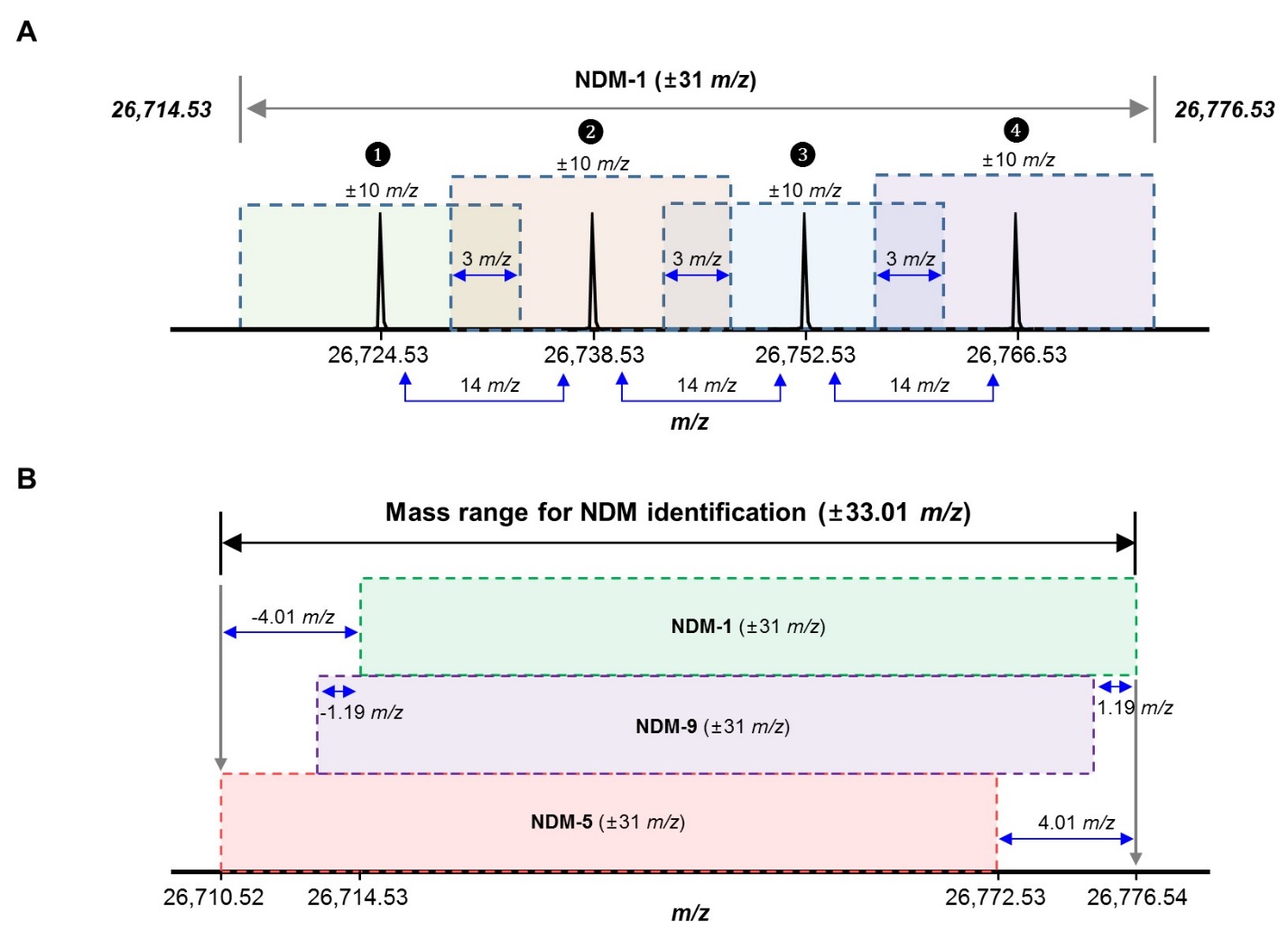
**

**Fig. S6. Mass range determination for NDM-1 proteoforms and NDM identification**

**A**, The masses are plotted with the average mass of each proteoform of NDM-1 protein after mass calculation using averagine. Each NDM-1 proteoform has +14 Da mass difference. Dashed boxes indicate the mass range for the identification of each proteoform (±10 *m/z*). Considering the overlaps of the ranges, the total mass range yields a ±31 *m/z* mass window for NDM-1 identification (26,714.53–26,776.53 *m/z*). **B**, The mass range for NDM (NDM-1, NDM-5, or NDM-9) identification. The range for NDM is from 26,710.52 *m/z* to 26,776.54 *m/z* (±33 *m/z*).


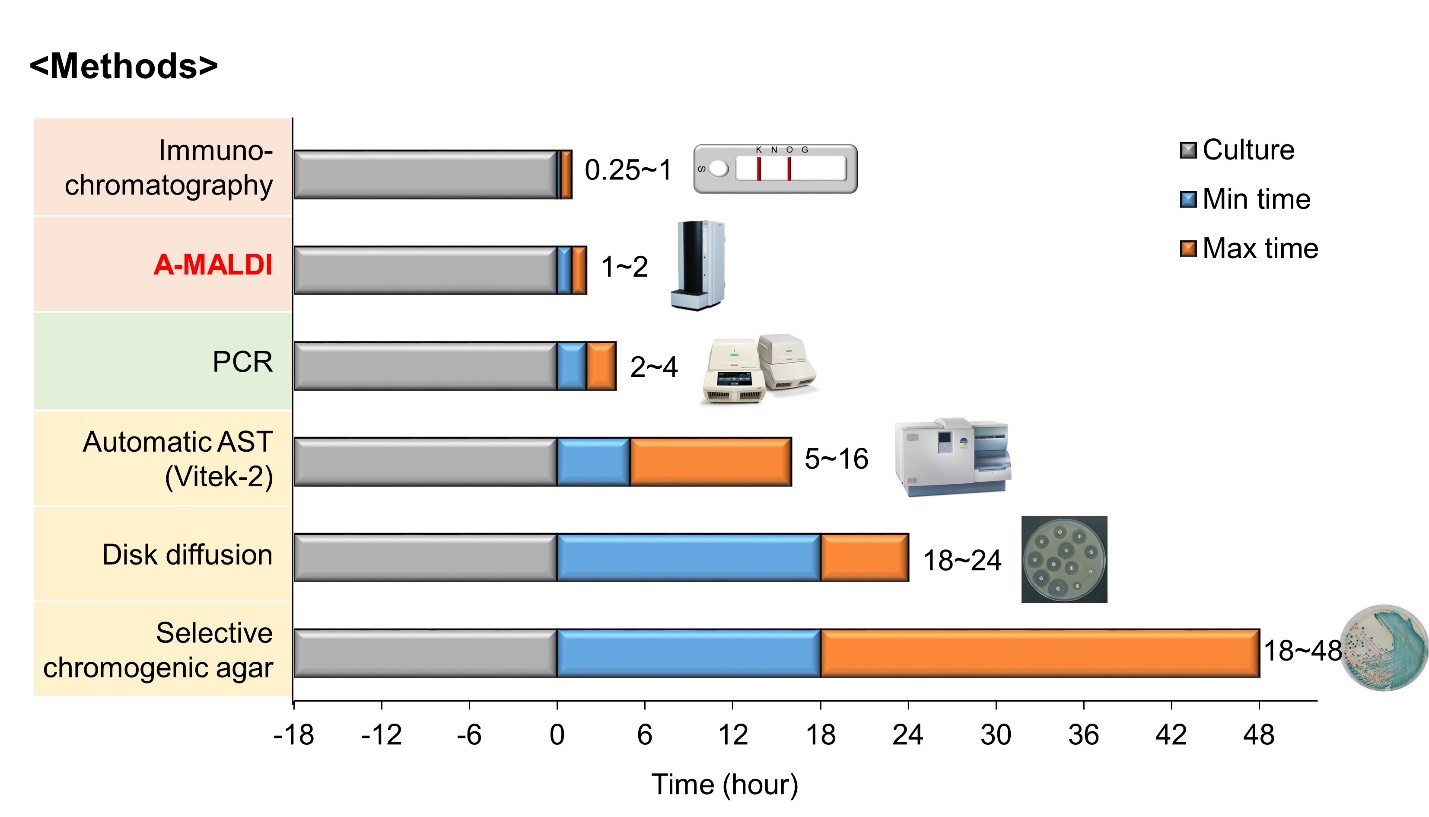


**Fig. S7. Comparison of traditional carbapenem-resistance testing methods and A-MALDI based on turnaround time**

The methods described above include immunochromatography, A-MALDI, PCR, automatic AST using Vitek-2, disk diffusion, and selective chromogenic agar. The methods highlighted in red boxes on the left represent protein-based assays, green boxes on the left indicate genetic-based assays, and yellow boxes on the left denote phenotype-based assays. The bars on the right represent the time span required for bacterial culture (grey), minimum processing time (blue), and maximum processing time (orange) for each method.


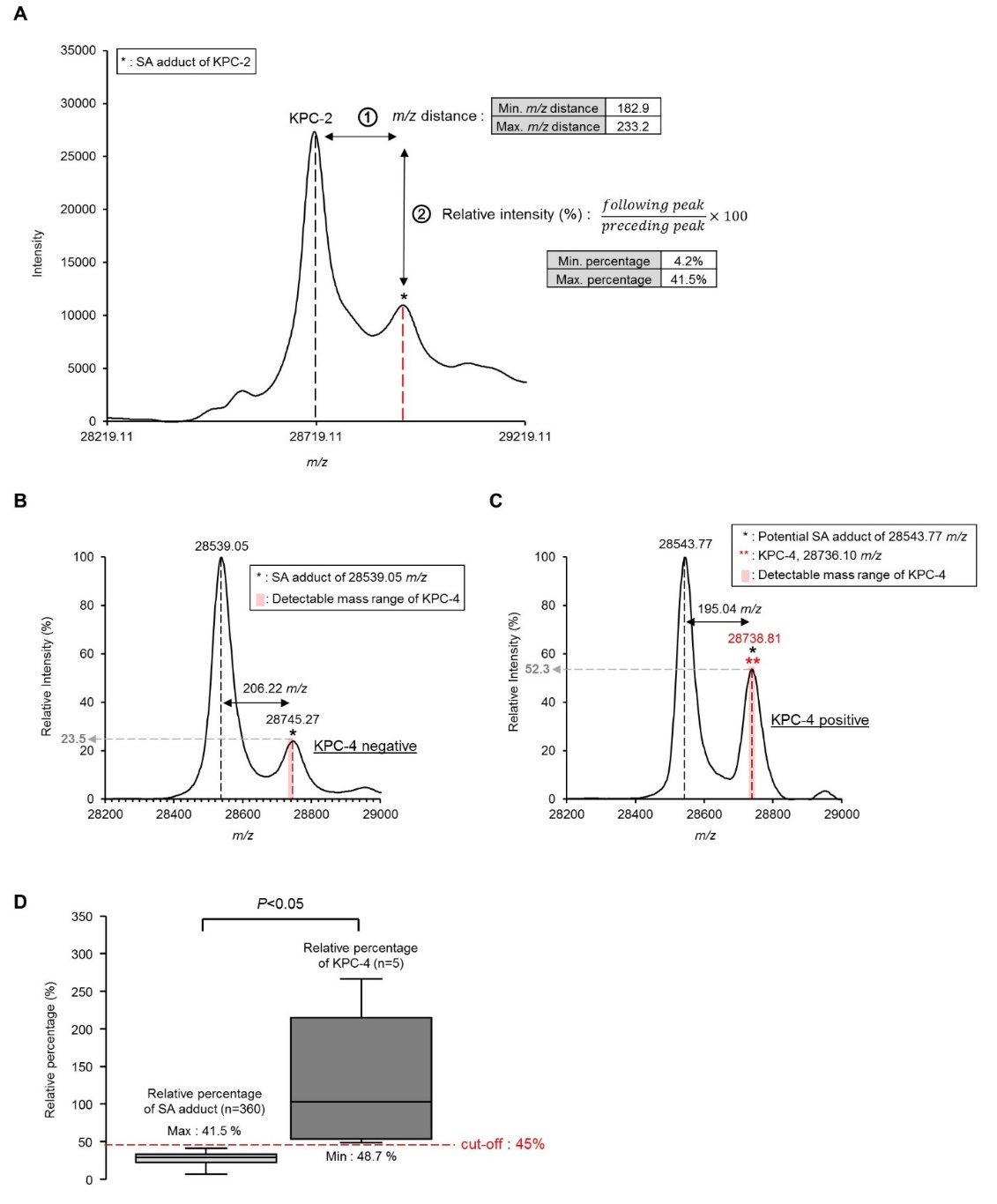


**Fig. S8.** **Determination of KPC-4 from the ambiguous peak caused by SA adduct**

**A,** Analysis of a spectrum with a KPC-2 peak and a SA adduct peak. The spectrum displays the peak of KPC-2 protein and the subsequent peak of SA adduct (black asterisk). The molecular weight difference between KPC-2 and the adduct peak is in the range of 182.9 *m/z* to 233.2 *m/z*, and the relative intensity percentage was 4.2–41.5%. **B,** Representative spectrum of a 28539.05 *m/z* protein peak and the subsequent SA adduct peak (black asterisk), where the SA adduct peak accounts for 23.5% of the intensity of the preceding protein peak. **C,** Representative spectrum of a 28543.77 *m/z* protein peak co-detected with the potential SA adduct or KPC-4 protein (black and red asterisks), where the KPC-4 peak and the preceding protein peak show an intensity ratio of 52.3%. **D,** Comparison of the relative ratio pattern of the SA adduct peak and the KPC-4 peak with SA adduct peak. While the adduct peak does not exceed a maximum value of 41.5%, the KPC-4 peak shows a minimum ratio of 48.7% (*P*<0.05, one-tailed Student's *t*-test).

**Table S1. Distribution of CPE genes by species**

| **Species (10)** |  |  |  |  |  |  |  | **CPE^a^** |  |  |  |  |  | **Non-CP-CRE** |
| --- | --- | --- | --- | --- | --- | --- | --- | --- | --- | --- | --- | --- | --- | --- |
|  | ***bla*_KPC_** | |  | ***bla*_NDM_** | | |  | ***bla*_OXA_** | | |  | ***bla*_GES_** | |  |
|  | **KPC-2** | **KPC-4** |  | **NDM-1** | **NDM-5** | **NDM-9** |  | **OXA-48** | **OXA-181** | **OXA-232** |  | **GES-5** | **GES-24** |  |
| *K. pneumoniae* | 281 | 5 |  | 11 |  |  |  | 1 | 19 | 6 |  |  |  | 52 |
| *E. coli* | 60 |  |  | 4 | 16 | 9 |  |  | 12 |  |  |  |  | 58 |
| *C. koseri* | 27 |  |  | 1 |  |  |  |  |  |  |  |  | 1 |  |
| *K. aerogenes* | 4 |  |  | 1 |  |  |  |  | 2 |  |  |  |  | 2 |
| *K. variicola* | 7 |  |  |  |  |  |  |  |  |  |  |  |  |  |
| *E. cloacae* | 1 |  |  | 4 |  |  |  |  |  |  |  | 1 |  |  |
| *C. freundii* | 1 |  |  | 2 |  |  |  |  |  |  |  |  |  |  |
| *K. oxytoca* | 1 |  |  | 2 |  |  |  |  |  |  |  |  |  |  |
| *M. morganii* | 1 |  |  |  |  |  |  |  |  |  |  |  |  |  |
| *E. asburiae* |  |  |  | 1 |  |  |  |  |  |  |  |  |  |  |
| Total | 383 | 5 |  | 26 | 16 | 9 |  | 1 | 33 | 6 |  | 1 | 1 | 112 |

^a^Confirmed by PCR/ DNA sequencing

1. Wang J, Wang H, Cai K, Yu P, Liu Y, Zhao G, Chen R, Xu R, Yu M. 2021. Evaluation of three sample preparation methods for the identification of clinical strains by using two MALDI-TOF MS systems. J Mass Spectrom 56:e4696.

2. Cheon DH, Lee S, Yang WS, Hwang S, Jang H, Kim MJ, Baek J-H. 2021. Optimization of a lysis method to isolate periplasmic proteins from Gram-negative bacteria for clinical mass spectrometry. PROTEOMICS – Clinical Applications 15:2100044.

3. Baek J-H, Rubinstein M, Scheuer T, Trimmer JS. 2014. Reciprocal changes in phosphorylation and methylation of mammalian brain sodium channels in response to seizures. Journal of Biological Chemistry 289:15363-15373.

4. Donnelly DP, Rawlins CM, Dehart CJ, Fornelli L, Schachner LF, Lin Z, Lippens JL, Aluri KC, Sarin R, Chen B, Lantz C, Jung W, Johnson KR, Koller A, Wolff JJ, Campuzano IDG, Auclair JR, Ivanov AR, Whitelegge JP, Paša-Tolić L, Chamot-Rooke J, Danis PO, Smith LM, Tsybin YO, Loo JA, Ge Y, Kelleher NL, Agar JN. 2019. Best practices and benchmarks for intact protein analysis for top-down mass spectrometry. Nature Methods 16:587-594.

5. Senko MW, Beu SC, McLaffertycor FW. 1995. Determination of monoisotopic masses and ion populations for large biomolecules from resolved isotopic distributions. J Am Soc Mass Spectrom 6:229-33.

6. González LJ, Bahr G, Nakashige TG, Nolan EM, Bonomo RA, Vila AJ. 2016. Membrane anchoring stabilizes and favors secretion of New Delhi metallo-β-lactamase. Nature Chemical Biology 12:516-522..

7. Randolph CE, Beveridge CH, Iyer S, Blanksby SJ, McLuckey SA, Chopra G. 2022. Identification of Monomethyl Branched-Chain Lipids by a Combination of Liquid Chromatography Tandem Mass Spectrometry and Charge-Switching Chemistries. Journal of the American Society for Mass Spectrometry 33:2156-2164.

8. Fagerquist CK, Garbus BR, Williams KE, Bates AH, Harden LA. 2010. Covalent attachment and dissociative loss of sinapinic acid to/from cysteine-containing proteins from bacterial cell lysates analyzed by MALDI-TOF-TOF mass spectrometry. J Am Soc Mass Spectrom 21:819-32.
